# Impact of Age Views on Cognition: Experimental, Longitudinal, and Ecological Momentary Findings

**DOI:** 10.1093/geroni/igaa057.2006

**Published:** 2020-12-16

**Authors:** Hans-Werner Wahl, Becca Levy, Brad Meisner, Andrea Gröppel-Klein, Deidre Robertson, Serena Sabatini, Anna Lücke

**Affiliations:** 1 University of Heidelberg, Heidelberg, Baden-Wurttemberg, Germany; 2 Yale School of Public Health, Woodbridge, Connecticut, United States; 3 York University, North York, Ontario, Canada; 4 University of Saarland, Saarbrücken, Saarland, Germany; 5 Economic and Social Research Institute, Dublin, Ireland; 6 University of Exeter, Exeter, England, United Kingdom; 7 Heidelberg University, Heidelberg, Baden-Wurttemberg, Germany

## Abstract

Research on the impact of age views on cognition has seen a strong momentum in recent years, fitting the stereotype embodiment theory prediction that the stereotypes taken in from a culture can impact older persons‘ cognition. These studies utilize experimental, longitudinal, and ecological momentary assessments (EMA), as well as a wide reach of cognitive outcomes. This symposium starts with two experimental studies. One demonstrates that negative age stereotypes reduce cognitive processing in older consumers (Gröppel-Klein et al.). A second study strives to better understand the pathway by which age stereotypes influence cognitive outcomes by focusing on dysregulation of reward-seeking behaviors and the downregulation of the dopaminergic system (Robertson et al.). We next explore two longitudinal studies that reveal differential relations among views of aging and various cognitive indicators. The first study found that older persons with more positive age beliefs are less likely to develop dementia even in a high-risk gene subpopulation of older adults (Levy et al.). The second study examined the association between awareness of age-related changes and cognitive scores (Sabatini et al.) Finally, Lücke et al. examine in their EMA study with 6 measurement occasions per day across 7 days that such a fine-tuned seems not to clearly support a linkage among subjective age and working memory for which beginning but not consistent evidence has been reported previously. Brad Meisner will discuss contributions in the light of meta-analytic finding revealing that older persons‘ negative age stereotypes can impair whereas their positive age stereotypes can improve cognitive performance.

